# Neutrophil CD64 can be an early predictor for sepsis during febrile neutropenic episodes in children with cancer: a case control study

**DOI:** 10.1186/s13052-025-01979-9

**Published:** 2025-05-14

**Authors:** Marwa Zakaria, Nehad Karam, Tamer Hassan, Nahla Zidan, Asmaa Abdelsalam, Raghdaa A. Ramadan, Marwa L. M. Rashad, Eman Abdelaziz, Ahmed Ramadan, Ahmed A. Ali

**Affiliations:** 1 Pediatric Department, Faculty of Medicine, Zagazig University, Zagazig, Egypt; 2 Clinical Pathology Department, Faculty of Medicine, Zagazig University, Zagazig, Egypt; 3 Microbiology Department, Faculty of Medicine, Zagazig University, Zagazig, Egypt

**Keywords:** Febrile neutropenia, Neutrophil CD64, Cancer, Sepsis

## Abstract

**Background:**

Febrile neutropenia (FN) is a common treatment-related complication in pediatric cancer patients with substantial morbidities and mortalities. Previous studies reported that neutrophil CD64 (n CD64) had higher diagnostic accuracy for infection with high sensitivity and specificity in neonates, pediatrics and adult patients. We aimed to evaluate the usefulness of neutrophil CD64 expression as an early diagnostic marker of sepsis in children with cancer during episodes of FN.

**Methods:**

a case control study was conducted on 100 children (50 patients with hematological malignancies and febrile neutropenia, 25 patients with hematological malignancies without febrile neutropenia and 25 apparently healthy children as a control group). Routine laboratory investigations including blood culture were done in patients with cancer according to our local standards. Procalcitonin level and Neutrophil CD64 expression by flowcytometry were measured for all study participants.

**Results:**

n CD64 expression was significantly higher in patients with cancer and FN compared to other groups (*p* > 0.001). At a cutoff value of ≥ 17.82%, serum n CD64 had 94% sensitivity and 72% specificity. n CD64 expression level was negatively correlated to absolute neutrophil count (ANC) during episode of FN (r= (-0.359, *p* = 0.01). A positive correlation was found between nCD64 expression and both of CRP and procalcitonin. Blood culture was positive in 54% in patients with cancer and FN. The most common isolated organism was Kllibselia pneumonia. Among patients with cancer and FN, n CD64 expression level was significantly higher in patients with positive blood culture compared to those with negative cultures.

**Conclusion:**

Neutrophil CD64 expression seems to be a reliable marker in early detection of sepsis during episodes of febrile neutropenia in children with hematological malignancies.

## Background

Febrile neutropenia (FN) is defined as an absolute neutrophil count (ANC) < 500 cells/µl or expected to decline to this level in the presence of an oral temperature > 38.3 °C or > 38.0 °C for one hour. Severe sepsis and septic shock in the setting of FN have been estimated to be 20–30% and 5–10% respectively [1].

Patients with hematological malignancies have higher risk for infection than patients with solid tumors. Early diagnosis of sepsis in children with febrile neutropenia and cancer still remains a challenge because of lack of specific laboratory markers as well as paucity of clinical signs that indicate bacterial infection in many cases of FN [2].

Blood culture, the gold standard of infection diagnosis, often takes 3–5 days and is limited by the relatively low positive rate, which restricts its use in early diagnosis [3]. Therefore, a new rapid and sensitive marker is needed for early diagnosis of bacterial infections in FN. Neutrophil CD64 is a membrane glycoprotein that mediates endocytosis, phagocytosis, antibody-dependent cellular cytotoxicity, cytokine release, and superoxide production. It is normally expressed on the surfaces of monocytes and macrophages [4].

The neutrophil CD64 expression has been investigated for years as a biomarker of infection and sepsis, given its reported low baseline expression and quick increase after inflammation [5]. Although most studies have focused on neonates and adults, only few studies have focused on its diagnostic value in children with cancer during the episodes of FN. In this work, we aimed to evaluate the usefulness of neutrophil CD64 expression as an early diagnostic marker of sepsis in children with cancer during episodes of FN.

## Patients and methods

This case control study was carried out at pediatric oncology unit and pediatric oncology outpatient clinic of Zagazig university hospitals, during the period from December 2021 to June 2022. The study was conducted on 100 children: 50 patients with hematological malignancies and febrile neutropenia (group 1), 25 patients with hematological malignancies without febrile neutropenia (group 2) and 25 apparently healthy children as a control group (group 3).

Patients were considered eligible for the study if they met the following inclusion criteria:

- Approval to sign an informed written consent.
- Age > 1year and < 18 years old.
- Children with hematological malignancies during febrile neutropenic episodes (for group 1).
- Children with hematological malignancies without febrile neutropenia (for group 2).

Patients were consecutively enrolled for the study on the basis of standard clinical, hematological, immunophenotypic and cytogenetic criteria for diagnosis of hematological malignancies and on the basis of the criteria of infectious diseases society of America (IDSA) for definition of febrile neutropenia.

Definition of febrile neutropenia (FN): is defined as an absolute neutrophil count (ANC) < 500 cells/µl or expected to decline to this level in the presence of an oral temperature > 38.3 °C or > 38.0 °C for one hour [1].

## Methods

Patients were subjected to full history taking, thorough clinical examination, routine laboratory investigations (including complete blood count and CRP initially and during febrile neutropenic episodes for group 1), radiological studies (including pelvi- abdominal ultrasound and CT chest when indicated) and specific laboratory investigations (including neutrophil CD64 expression by flowcytometry).

## Sample collection

Eight mL of venous blood sample were withdrawn from each subject by venipuncture and delivered into the following tubes: one mL was delivered into EDTA vacutainer tube for CBC, two mL were delivered into sterile plain vacutainer tubes for CRP and PCT analysis, Immunophenotyping by Flowcytometry for detection of CD64 expression was performed using the same sample for CBC examination. Samples were processed within two hours from collection. and five mL were used for blood culture. Blood samples were withdrawn after the onset of fever and before the start of antibiotic therapy in febrile patients.

Complete blood count was performed using Sysmex XN1000 automated cell counter (Sysmex, Japan), CRP was done on Cobas 6000 autoanalyzer (Roche diagnostics, Germany) and serum PCT levels were detected by a chemiluminescence sandwich immunoassay on Cobas 6000 autoanalyzer (Roche diagnostics, Germany).

Immunophenotyping and detection of CD64 expression by flowcytometry: Multicolor flow cytometry for immunophenotyping (BD FACSCantoTM II flow Cytometry, Becton Dickinson, San Jose, USA) was done to measure expression of neutrophil CD64.

## Results

The mean age of patients in group 1 was 7.12 years. They were 29 (58%) males and 21 (42%) females. 54% of them had acute lymphoblastic leukemia, 28% had acute myeloid leukemia and 18% had lymphoblastic lymphoma. In group 2, the mean age of patients was 6.84 years. They were 12 (48%) males and 13 (52%) females. 56.0% of them had acute lymphoblastic leukemia, 32% had acute myeloid leukemia and 12% had lymphoblastic lymphoma. In group 3, the mean age of patients was 7.84 years. They were 11 (44%) males and 14 (56%) females. Age and sex of studied groups as well as type of hematological malignancy were displayed in Table 1.

**Table 1:** Age and sex of the studied groups

	**Group1 (cancer with FN) (** ***n*** ** = 50)**	**Group 2 (cancer without FN) (** ***n*** ** = 25)**	**Group 3 (healthy controls) (** ***n*** ** = 25)**	**Test**	***P***
**Age (years)**					
Min.– Max.	1–16	1–17.	1–17	H = 0.6	0.7
Mean ± SD.	7.12 ± 4.28	6.84 ± 3.9	7.84 ± 4.4		
**Sex** **(n,5)**					
Male	29 (58%)	12(48%)	11(44%)	χ^2^ = 1.5	0.47
Female	21(52%)	13(52%)	14 (56%)		
**Type of malignancy (*n***, **%)**	**Group1 (cancer with FN) (** ***n*** ** = 50)**	**Group 2 (cancer without FN) (** ***n*** ** = 25)**			
ALL	27 (54%)	14 (56%)		χ2 = 33.758	< 0.001
AML	14 (28%)	8 (32%)			
Lymphoblastic lymphoma	9 (18%)	3 (12%)			

χ2: chi-square test. H: Kruskal-Wallis test. SD: Standard Deviation

Regarding treatment phase, 82% of patients in group 1 were in the induction phase and 18% were in the consolidation phase while in group 2, all patients were in induction phase. As regards clinical presentation in group 1, organomegaly was present in 80% of patients, tachycardia in 42%, abdominal pain in 42%, vomiting in 38%, tachypnea in 36%, cough in 20%, lower limb oedema in 16%, diarrhea in 14%, convulsions in 10% and headache in 10%. CT chest revealed chest infection in 20% of patients and abdominal ultrasound showed organomegaly in 92% of patients in group 1.

In group 1, blood culture was positive in 27 patients (54%). 15 patients (55.5%) were positive for gram negative organisms. The most common isolated gram-negative organisms were Klebsiella pneumonia in 7 patients (26%), pseudomonas aeruginosa in 6 patients (22%) and E-coli in 2 patients (7%). 12 (44.5%) patients were positive for gram positive organisms. The most common isolated gram-positive organisms were Staph aureus in 8 patients (30%), coagulase negative staph in 3 patients (11%) and Strept viridans in one patient (4%). The median expression level of nCD64 in patients with positive blood culture was 31.4 versus 27.2 in those with negative blood culture. Results of blood culture were represented in Table 2.

**Table 2:** Results of blood culture in cancer patients with FN

Blood culture	*N* (50)	%
**Negative**	23	46.0
**Positive**	27	54.0
Gram -ve	15	55.5
Gram + ve	12	44.5
** Isolated organisms**	**N (27)**	
**Gram negative**		
Klebsiella pneumonia	7	26%
Pseudomonas aeruginosa	6	22%
E. coli	2	7%
**Gram positive**		
Staph aureus	8	30%
Coagulase negative staph	3	11%
Strept viridians	1	4%

The median expression level of nCD64 was significantly higher in group 1 compared to group 2 and group 3 (28.71% versus 16.65% and 12.57% respectively, *P* < 0.001). also, when comparing the median expression of nCD64 level in febrile patients with positive blood culture versus group 2 and group 3 it was 31.4 versus 16.65% and 12.57% respectively, *P* < 0.001). As regards, C reactive protein and procalcitonin blood levels, there was highly significant difference in group 1 compared to group 2 and group 3 (56.45 mg/dl versus 5.6 mg/dl and 3.0 mg/dl respectively for CRP and 0.44ng/dl versus 0.09ng/dl and 0.06 ng/dl respectively for procalcitonin (Table 3).

**Table 3:** Serum levels of nCD64, CRP and procalcitonin in studied groups

	Group1 (cancer with FN) (*n* = 50)	Group 2 (cancer without FN) (*n* = 25)	Group 3 (healthy controls) ( *n* = 25)	Test	*P*
**nCD64 (%)**					
Min.– Max.	16.03–63.12	6.82–36.67	7.08–29.15	H = 53.15	< 0.001
Median (IQR)	28.71(25.38–34.81)	16.65 (10.89–25.12)	12.57 (9.25–14.71)		
Posthoc test	P1 < 0.001	P2 = 0.115	P3 < 0.001		
**C-Reactive protein (CRP)**					
Min.– Max.	3.0–280.0	3.0–9.0	2.9–9.0	H = 64	< 0.001
Median (IQR)	56.45(15.45–129.63)	5.6 (3.05–6.0)	3.0(3.0–3.75)		
Pairwise test	P1 < 0.001	P2 = 0.299	P3 < 0.001**		
**Procalcitonin(ng/ml)**					
Negative (< 0.5) Positive (> 0.5)	33 (66.0%) 17 (34.0%)	20 (80.0%) 5 (20.0%)	25 (100.0%) 0 (0.0%)	χ2 = 11.3	0.004
Min.– Max. Median (IQR)	0.02–14.77 0.44 (0.11–7.03)	0.02–0.81 0.09 (0.04–0.34)	0.03–0.39 0.06(0.04–0.1)	H = 20.43	< 0.001
Pairwise	P1 = 0.005	P2 = 0.212	P3 < 0.001		

H: Kruskal-Wallis test. χ2: chi-square test. IQR: interquartile range

**Fig. 1:**
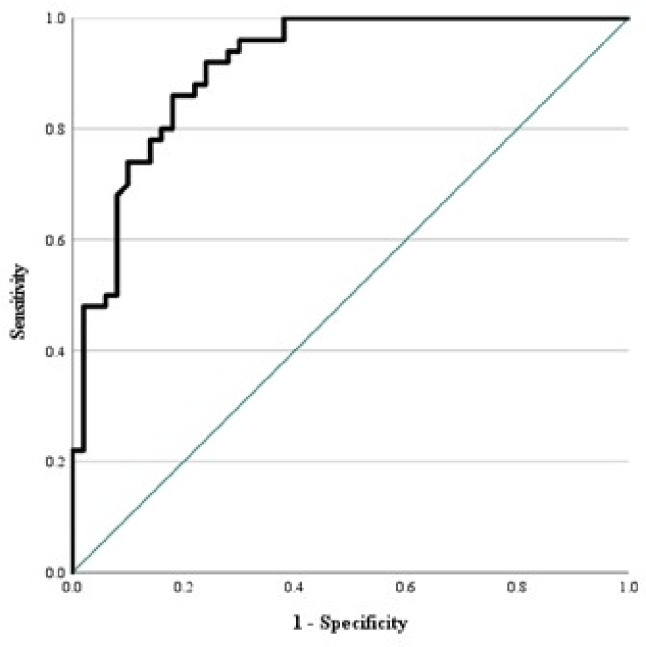
ROC curve for nCD64 to discriminate patients group from control group

The best cutoff for expression level of nCD64 in prediction of febrile neutropenia among patients of cancer was ≥ 17.82% with area under curve 0.913, 94% sensitivity, 72% specificity, 77% positive predictive value, 92.3% negative predictive value. (Table 4; Fig. 1)

**Table 4:** Area under the curve, sensitivity, specificity for nCD64, CRP and procalcitonin

	AUC	*p*	95% C.I	Cut off^#^	Sensitivity	Specificity	PPV	NPV
nCD64( %)	0.913	< 0.001	0.858–0.968	≥ 17.82	94.0	72.0	77.0	92.3
CRP(mg/dl)	0.756	< 0.001	0.659–0.852	≥ 14.85	73.5%	68.6%	69.2%	69.2%
Procalcitonin (ng/ml)	0.752	< 0.001	0.492–0.724	> 0.091	80%	60%	66.7%	75%

AUC: area under the curve. PPV: positive predictive value. NPV: negative predictive value

At a cut off level of ≥ 6.5 mg/dl, CRP had a diagnostic performance to discriminate patients’ group from control group with statistically significant difference and at a cut off level of ≥ 0.091 ng/ml, Procalcitonin had a diagnostic performance to discriminate patients’ group from control group with statistically significant difference (Table 4).

Comparing the ROC curves of the three parameters revealed that there were significant differences (*P* < 0.001) between AUC of nCD64 expression (0.913) when compared to that of CRP (0.756) and PCT (0.752) (Table 4).

The mean nCD64 expression level was significantly higher in patients with positive blood culture (*p* = 0.01), positive CT chest findings (0.03) and positive procalcitonin (*P* = 0.02) (Table 5). Moreover, nCD64 expression was negatively correlated with absolute neutrophil count (*r* = − 0.359, *P* = 0.01) while nCD64 expression was positively correlated with CRP (*r* = 0.330, *p* = 0.02) and Procalcitonin level (*r* = 0.410, *p* = 0.003) (Table 6).

**Table 5:** Relationship between nCD64 and different parameters in cancer patients with FN

	No.	nCD64 (%)		Mann Whitney test	*p*
		Mean ± SD	Median (Min.– Max.)		
**CT chest (Pneumonia)**					
No	40	29.77 ± 8.85	27.54 (16.03–52.64)	U = 111.5	0.03
Yes	10	40.77 ± 15.66	31.71 (25.79–63.12)		
**PAUS (Hepatosplenomegaly)**					
No	4	36.55 ± 15.28	31.15 (25.26–58.63)	U = 75.0	0.57
Yes	46	31.57 ± 11.00	28.71 (16.03–63.12)		
**Blood culture**					
Negative	23	27.88 ± 8.72	26.03 (16.03–52.64)	U = 183.50	0.01
Positive	27	35.45 ± 12.17	33.12 (20.49–63.12)		
**Procalcitonin (ng/ml)**					
Negative (< 0.5)	33	28.87 ± 8.38	27.60 (16.03–49.78)	U = 161.50	0.02
Positive (> 0.5)	17	37.97 ± 13.82	33.14 (21.54–63.12)		

CT: Computed Tomography; PAUS: Pelvi-abdominal Ultrasound; SD: Standard Deviation; U: Mann Whitney test

**Table 6:** Correlation between nCD64 with different parameters in patients group

nCD64	*r*	*p*
Age (years)	-0.205	0.153
Hemoglobin (g/dl) during FN	0.092	0.524
Platelets (×10^3^/ul) during FN	-0.064	0.660
TLC (×10^3^/ul) during FN	0.159	0.269
ANC (×10^3^/ul) during FN	-0.359	0.01
CRP(mg/dl)	0.330	0.02
Procalcitonin (ng/ml)	0.410	0.003

r: correlation coefficient

## Discussion

Febrile neutropenia in children treated for malignancy is a common and direct sequela of chemotherapy. Episodes of FN can be life- threatening, and demand prompt recognition, assessment and treatment with broad spectrum antibiotics. While in the majority of episodes no causal infection is identified, 30% are secondary to documented infection [6].

A rapid laboratory test with high specificity for pediatric sepsis would be a valuable tool in therapeutic decision making and avoiding the unnecessary use of antibiotics.CD64 is mainly involved in phagocytosis and intracellular killing of pathogens, but it is also expressed at very low levels on the surface of unstimulated neutrophils. Upregulation of CD64 on neutrophils is thought to be a very early step of host’s immune response to bacterial infection, increasing approximately one hour after invasion [7].

CD64 has several desirable biomarker characteristics and can be used for differentiating bacterial infection from other inflammatory disorders [8]. However, the reliability of nCD64 expression in FN has not been well demonstrated so far. In this study we aimed to evaluate the significance of nCD64 expression in diagnosis of bacterial infection in children with hematological malignancies during the episodes of FN.

In our study, acute lymphocytic leukemia (ALL) was the commonest hematological malignancy (54%) followed by acute myeloid leukemia (AML) (28%) and lymphoblastic lymphoma (LBL) (18%). These results are in agreement with previous studies on hematological malignancies. Horibe et al. reported in their study that ALL had the highest incidence (46.6%), followed by AML (16.7%) and non-Hodgkin lymphoma (NHL, 11.9%) [9]. Also, Badiee et al., showed that the most common hematological malignancies were ALL (58%), followed by AML (24%) [10] These data were also supported by Urbonas et al. [11].

In the current study, 54% of the patients had positive blood cultures and 46% had negative blood culture. Similarly, Efe İris et al., reported that among the 31 episodes of FN, 17 episodes revealed positive blood culture and 14 episodes revealed negative blood [12]. On the contrary, Barbosa et al., found that 21% of the studied subjects had positive blood culture and 79% had negative blood culture [13]. Variability of results may be attributed to administration of antibiotics 48 h before sample withdrawal, diagnostic workup may be insufficient or incomplete or sepsis caused by unusual organisms that are difficult to be identified in routine practice [14].

Among patients with positive blood culture, 15 (55.5%) were positive for gram negative organisms and 12 (44.5%) were positive for gram positive organisms.

Our results were matched with those reported by Lima et al., where gram-negative pathogens were more frequently isolated (49.6%), followed by gram-positive pathogens (41.2%) [15]. Similarly, Jacob et al., found that 56.25% of the positive cultures yielded gram-negative bacteria and 31.25% yielded gram-positive bacteria [16]. On the contrary, Lehrnbecher et al., reported that Blood stream infections occurred in 88% 228 episodes (Gram-positive (*n* = 202) and Gram-negative (*n* = 42) pathogens) [17]. Wisplinghoff et al., showed in their study that gram positive pathogens were more predominant than gram negative pathogens 76% versus 14% respectively [18]. Viscoli et al., and Castagnola et al., reported the predominance of gram-positive pathogens over gram negative pathogens and they attributed these findings to treatment for cancer has become more intensive and associated with severe oral mucositis and diarrhea, leading to major damage of mucosal barriers and an increased risk of infection due to resident gram-positive oral flora. In addition, patients with cancer are fitted with partially or totally implantable intravenous catheters more often than in the past, a fact that might explain the increasing number of staphylococcal infections [19, 20].

The most common isolated gram-negative organisms were Klebsiella pneumonia in 7 patients (47%), pseudomonas aeruginosa in 6 patients (40%) and E-coli in 2 patients (13%). In agreement of our results, Worku et al., reported that the K. pneumonia and P. aeruginosa were the most common gram-negative isolated organisms among cancer patients (47% and 29.5% respectively) [21]. On the contrary, Stergiotis et al., found that E. coli was more common than Pseudomonas aeruginosa and Klebsiella (17%, 9.5% and 5% respectively) [22].

The most common isolated gram-positive organisms were Staph areus in 8 patients (66.7%), coagulase negative staph in 3 patients (25%) and Strept viridans in one patient (8.3%). These results were in line with those reported by Jacob et al., where staphylococcus aureus was the most common gram-positive organism isolated in FN patients [14]. The same was reported by Siddiqui et al., where the frequency of staphylococcus aureus was 16% [23]. Stergiotis et al., found that Coagulase-negative staphylococci (32%) and strept viridans group (22%) were the most common isolated gram-positive organisms [22].

Our results showed that cancer patients with FN had significantly higher expression level of nCD64 compared to cancer patients without FN and control group (median nCD64 expression level was 28.71% versus 16.65% and 12.57% respectively, *P* = 0.001). Similarly, Liang et al., (2021), reported that the nCD64 expression level was significantly elevated in neutropenic children with confirmed infection status (*P* < 0.05) [24]. García-Salido et al., found that nCD64 expression on a blood sample at PICU admission was higher in case of bacterial infection (*P* = 0.001) [25].

Also, in our study, cancer patients with FN had significantly higher blood level of CRP and procalcitonin compared to the other groups (56.45 mg/dl versus 5.6 mg/dl and 3.0 mg/dl respectively for CRP and 0.44ng/dl versus 0.09ng/dl and 0.06 ng/dl respectively for procalcitonin.

Our results were matched with Mohamed et al., where they found highly significant increase in CRP in patients with culture proven sepsis group compared to the clinically diagnosed sepsis group (44.69 ± 31.57 versus 25.14 ± 14.15, *p* = 0.01) [26]. In an earlier study conducted by Rintala et al., high CRP levels were associated with sepsis and microbiologically documented infection in patients with FN (*p* = 0.002) [27]. Also, van der et al., and García-Salido et al., reported that PCT level was significantly elevated in patients with bacterial infection compared to patients without bacterial infection [8, 25].

On the contrary, Aimoto et al., reported that PCT levels were not useful tool in sepsis. This negative result may be explained in part by the definition of infection or the sampling time where sampling time falling within one day of sepsis onset might lead to diagnostic performance being underestimated [28]. Also, Ebihara et al., reported that there were no significant differences in CRP levels between infected and non-infected groups in neutropenic patients (*p* = 0.62) [29]. There were some limitations in Ebihara et al., First, it was a retrospective analysis, and they were not able to obtain sufficient samples to measure the patients’ biomarker levels throughout the course of their fevers and the associated treatment [29].

In the current study, nCD64 expression was negatively correlated to absolute neutrophil count (ANC) (*r*=- 0.359, *P* = 0.01). This can be explained based on the fact that the severity of neutropenia (the lower the ANC) is directly correlated with the severity of infection (Meckler and Lindemulder, 2009) [30]. Similarly, liu et al., found that nCD64 expression level correlates directly with the severity of infection [31]. Our results showed that nCD64 expression was positively correlated with CRP (*r* = 0.330, *p* = 0.019) and procalcitonin level (*r* = 0.410, *p* = 0.003). Similarly, García-Salido et al., confirmed the positive correlation between the nCD64 expression and both CRP (*P* = 0.026, *r* = 0.352) and PCT (*P* = 0.001, *r* = 0.487) [25]. Also, Abd Elkareem et al., found a significant positive correlation between the nCD64 and CRP (*P* = 0.003, *r* = 0.4) [32].

The best cutoff level of nCD64 expression in prediction of febrile neutropenia among patients of cancer was ≥ 17.82% with area under curve 0.913, 94% sensitivity, 72% specificity, 77% positive predictive value, 92.3% negative predictive value and 83% overall accuracy. At a cut off level of ≥ 6.5 mg/dl, CRP had a diagnostic performance to discriminate patients’ group from control group with statistically significant difference and at a cut off level of ≥ 0.091 ng/ml, Procalcitonin had a diagnostic performance to discriminate patients’ group from control group with statistically significant difference.

In our study, comparing the ROC curves of the three parameters revealed that there were significant differences (*P* < 0.001) between AUC of nCD64 expression (0.913) when compared to that of CRP (0.756) and PCT (0.752). It was concluded that the reliability ofnCD64 expression was higher than that of PCT and CRP in prediction of bacteremia in FN patients.

Similar findings were shown by Shang et al.,) who found that the area under the curve of the nCD64 expression was 0.777 with a sensitivity of 82.3% and a specificity of 67.2% and this was higher than that for PCT (0.735, sensitivity 67.8% and specificity 71.6%) and CRP (0.670, sensitivity 54.4% and specificity 74.6%). The positive and negative likelihood ratios were also better for the nCD64 than either PCT or CRP [33]. Jiabao et al., reported that nCD64 expression was superior to PCT (AUC 0.844 and 0.599 respectively, Sensitivity76.2% and 50% respectively and specificity 71.4% and 63.4% respectively) [24]. Similarly, Ye et al., reported that nCD64 is a very powerful biomarker superior to PCT for the diagnosis of sepsis (AUC for nCD64 and PCT were 0.904 and 0.863, respectively) [7]. Also wang et al., suggested that the nCD64 is a helpful marker for early diagnosis of sepsis in critically ill patients with sensitivity88%, specificity90% and AUC 0.96 [34].

On the contrary, Barbosa et al., found a poor sensitivity and specificity of nCD6 (64.3% and 42% respectively) for detection of sepsis [13].

The different results may be attributed to different sampling time, study population, severity of neutropenia and severity of infection.

In our study, there was a significant relationship between nCD64 expression and positivity of blood culture (35.45% in patients with positive blood culture versus 27.88% in patients with negative blood culture). This relationship supported that the reliability of nCD64 expression to predict significant bacteremia and sepsis in febrile neutropenic patients. These results were matched with Icardi et al., and Efe İris et al., where they found that nCD64 index was a useful test for the detection of significant bacterial infection [12, 35].

## Conclusions

We concluded that nCD64 expression was superior to PCT and CRP in early detection of sepsis in patients with hematological malignancies during episodes of FN.

### Limitation of the study

Small sample size was one of the limitations in this study and so larger multicenter studies are still needed to support these findings.

## Data Availability

The data sets generated during and/or analyzed during the current study are available from the corresponding author on reasonable request.

## Abbreviations

n CD64: neutrophil CD64
FN: Febrile neutropenia
ANC: Absolute neutrophil count
IDSA: Infectious diseases society of America
PCT: Procalcitonin
CRP: C-reactive protein
CT: Computed tomography
ELISA: Enzyme linked immunosorbent assay
